# Percutaneous closure of residual leak after surgical left atrial appendage ligation: a case of stroke prevention in high-risk atrial fibrillation

**DOI:** 10.1186/s12872-026-05689-w

**Published:** 2026-03-06

**Authors:** Mert Doğan, Ahmet Hakan Ateş, Cem Çöteli, Uğur Nadir Karakulak, Hikmet Yorgun, Mehmet Levent Sahiner, Ergun Barıs Kaya, Kudret Aytemir

**Affiliations:** Faculty of Medicine, Department of Cardiology, Hacettepe University, Sihhiye, 06100 Ankara Turkey

## Abstract

Incomplete surgical left atrial appendage (LAA) closure is associated with increased thromboembolic risk in atrial fibrillation patients. We present a case of successful percutaneous closure of a significant residual leak following surgical LAA ligation. A 64-year-old female with atrial fibrillation (CHA₂DS₂-VASc score 4), prior surgical interventions including LAA ligation for thrombus formation, presented with ischemic stroke despite anticoagulation. Transesophageal echocardiography revealed a 10 mm residual communication following incomplete LAA closure. Percutaneous closure was performed using a 31 mm Amplatzer Amulet device with successful leak elimination. Percutaneous closure of residual leaks following incomplete surgical LAA closure represents a viable alternative to repeat surgery in high-risk patients. Individualized antithrombotic therapy is essential for optimal outcomes.

## Introduction

Left atrial appendage (LAA) exclusion during cardiac surgery has become an increasingly utilized strategy for stroke prevention in patients with atrial fibrillation (AF). The LAA is the source of over 90% of cardiac emboli in non-valvular AF patients [1]. However, incomplete surgical closure remains a significant challenge, occurring in 10–30% of cases [2, 3]. These residual communications may paradoxically increase thromboembolic risk compared to an unligated appendage, as the surgically altered anatomy promotes stasis and thrombus formation [4]. We present a case of successful percutaneous closure of a significant residual leak following surgical LAA ligation in a high-risk patient with recurrent stroke.

## Case

**Fig. 1:**
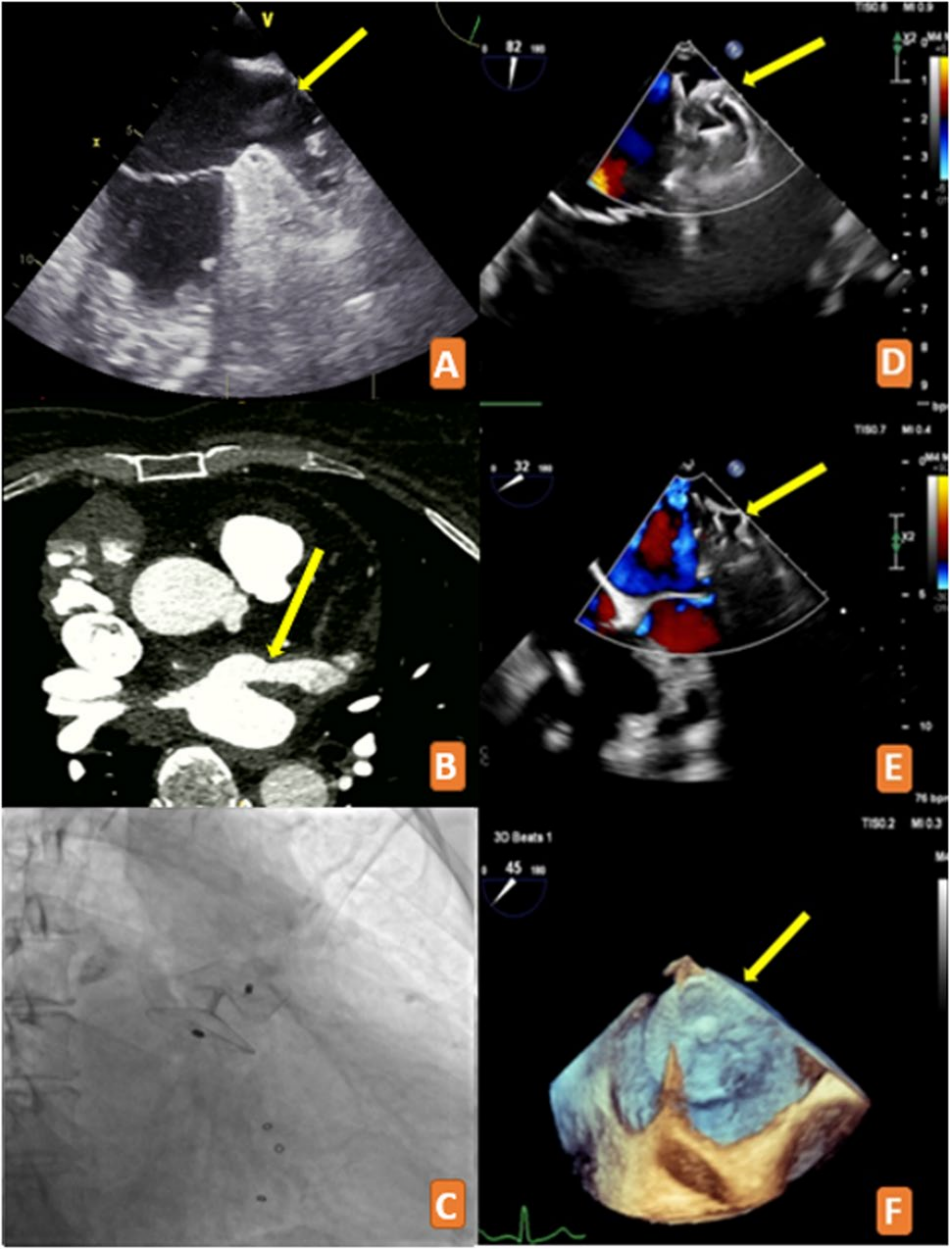
**A** Pre-surgical transesophageal echocardiography demonstrating thrombus formation at the left atrial appendage ostium. **B** Cardiac CTA demonstrating 10 mm residual left atrial appendage leak (arrow). **C** Fluoroscopic image (RAO cranial projection) showing successful device deployment with no contrast passage, confirming complete leak closure. **D** Transesophageal echocardiography with color Doppler demonstrating no flow passage through the device, indicating successful leak closure. **E** Two-dimensional transesophageal echocardiography at 2-month follow-up showing no leak passage. **F** Three-dimensional transesophageal echocardiography demonstrating stable device position

A 64-year-old female with a history of atrial fibrillation, hypertension, diabetes mellitus, and prior surgical interventions including bioprosthetic tricuspid valve replacement for tricuspid regurgitation, left atrial appendage surgical ligation for thrombus formation while on rivaroxaban, and MAZE procedure presented to our clinic with complaints of palpitations and dyspnea (Fig. 1 A). Physical examination revealed bilateral mild pretibial edema and minimal rales in the basal zones of the lungs. Her 12-lead electrocardiogram showed atrial fibrillation with a ventricular rate of 130 beats/min. The patient was receiving apixaban 5 mg BID for oral anticoagulation therapy. She had experienced an ischemic cerebrovascular event two months prior. The patient’s CHA₂DS₂-VASc score was calculated as 4. Laboratory findings showed normal creatinine levels and a BNP value of 133.99 pg/mL. Transthoracic echocardiography demonstrated an ejection fraction of 61%, mild mitral regurgitation, and normal function of the bioprosthetic tricuspid valve. Catheter AF ablation was planned for the patient. Transesophageal echocardiography (TEE) revealed a significant residual communication at the LAA closure site. Cardiac computed tomography angiography (CTA), performed as part of pre-ablation planning, confirmed and quantified the leak as 10 mm at its narrowest point using multiplanar reconstruction (MPR) in axial and coronal planes at the level of maximum narrowing between the LAA and left atrium (Fig. 1B). Due to the patient’s recent history of ischemic cerebrovascular event and previous thrombus in the LAA, percutaneous LAA leak closure was planned. Under general anesthesia, an 8 French sheath was placed in the right femoral vein. A 6 French sheath was inserted into the left femoral artery for invasive blood pressure monitoring. Using the right femoral vein approach, a delivery catheter was positioned in the left atrium following transseptal puncture from the inferoposterior region. With the assistance of an RF Marinr™ MC (Medtronic, USA) catheter, the delivery catheter was inserted through the LAA leak ostium into the LAA. Device selection was based on the preserved native LAA anatomy beyond the leak site, with the landing zone measuring 26 mm at its widest diameter. A 31 mm Amplatzer Amulet device (St. Jude Medical, St. Paul, MN, USA) was chosen to ensure adequate oversizing for stable deployment and complete leak closure (Fig. 1 C, D). The patient was discharged on dual therapy with apixaban 5 mg BID and aspirin 81 mg daily, planned for 3–6 months followed by apixaban monotherapy indefinitely, given her high thrombotic risk profile including prior stroke, CHA₂DS₂-VASc score of 4, and history of LAA thrombus formation. The patient returned for follow-up after two months with no complaints. TEE was performed to evaluate the LAA leak. No leak was observed, and the device remained stable in the appropriate position (Fig. 1E, F).

## Discussion

Surgical LAA ligation has been increasingly utilized as an adjunctive procedure during cardiac surgery to reduce thromboembolic risk in patients with atrial fibrillation. However, incomplete LAA closure or development of leaks following surgical ligation represents a significant clinical concern, occurring in 10–30% of cases according to recent studies [2, 3]. The pathophysiology of increased risk with incomplete closure is particularly concerning: the surgically altered anatomy creates a partially excluded pouch that promotes blood stasis and may be more thrombogenic than a completely patent appendage [4]. Studies have demonstrated that residual communications > 5 mm are associated with significantly increased stroke risk, with some reports showing stroke rates as high as 15% in patients with incomplete surgical closure [5, 6].

In our case, the presence of a 10 mm residual communication combined with the patient’s recent ischemic stroke despite adequate anticoagulation and high CHA₂DS₂-VASc score necessitated intervention. The decision to proceed with percutaneous closure rather than continued medical therapy alone or repeat surgical intervention was based on several factors: the patient’s demonstrated failure of anticoagulation (stroke while on therapy), the technical feasibility of percutaneous approach, and the desire to avoid the morbidity of repeat cardiac surgery.

Percutaneous closure of post-surgical LAA leaks has emerged as an effective treatment modality, with multiple devices successfully employed for this indication. Various devices have been used, including Amplatzer septal occluders, vascular plugs, and dedicated LAA closure devices [7, 8]. The Amplatzer Amulet device was selected in our case due to its dual-seal mechanism (lobe and disc design) which is particularly suitable for larger, tubular post-surgical defects, providing both anchoring within the preserved LAA structure and sealing at the ostium. The device sizing strategy differs from de novo LAA closure, as the landing zone within the preserved native LAA structure, rather than the leak diameter itself, determines the appropriate device size [9].

Evidence-based guidelines for antithrombotic therapy following percutaneous closure of post-surgical LAA leaks are lacking, reflecting the limited data available for this specific clinical scenario [10]. Current literature suggests individualizing therapy based on thrombotic and bleeding risk profiles [11]. While standard post-LAA closure protocols recommend short-term anticoagulation followed by antiplatelet therapy, our patient’s high-risk features (recent stroke, prior LAA thrombus, CHA₂DS₂-VASc score of 4) warranted continued anticoagulation. The combination of apixaban and aspirin for 3–6 months followed by apixaban monotherapy represents a balance between preventing device-related thrombosis during endothelialization and long-term stroke prevention [12].

An important consideration in our patient was the planned catheter ablation for AF. Studies have demonstrated the feasibility of catheter ablation following both surgical LAA ligation and percutaneous LAA closure [13, 14]. Walker and Phillips reported successful ablation in patients with pre-existing Watchman devices, achieving satisfactory LAA occlusion in 92% of patients [15]. Combined procedures of catheter ablation and LAA closure have shown excellent long-term outcomes with observed stroke rates as low as 0.5% per year during 5-year follow-up [16]. Recent advances with pulsed field ablation technology have shown that even LAA clips do not inhibit the pulse delivery, expanding ablation options for patients with LAA closure devices [17]. Furthermore, the safety of pulmonary vein isolation using pulsed field ablation in patients with implanted Watchman devices has been demonstrated, with no major adverse events reported [18]. The presence of the LAA closure device does not preclude subsequent ablation procedures, though it may limit access to the LAA for mapping and ablation of LAA triggers.

It is important to acknowledge that the evidence supporting percutaneous closure of post-surgical LAA leaks comes primarily from case series and observational studies. Randomized controlled trials comparing different management strategies for this specific clinical scenario are lacking. However, the growing body of experience suggests that percutaneous closure is a safe and effective alternative to repeat surgery, with success rates exceeding 95% in experienced centers [19].

## Data Availability

The data that support the findings of this study are available on request from the corresponding author.

## Abbreviations

AF: Atrial Fibrillation
LAA: Left Atrial Appendage
TEE: Transesophageal Echocardiography
CTA: Computed Tomography Angiography
MPR: Multiplanar Reconstruction
BNP: B-type Natriuretic Peptide
BID: Bis In Die (twice daily)
